# A rare case of nivolumab-related myasthenia gravis and myocarditis in a patient with metastatic gastric cancer

**DOI:** 10.1186/s12876-021-01904-4

**Published:** 2021-08-26

**Authors:** Masaru Komatsu, Motoharu Hirai, Kazuaki Kobayashi, Hideki Hashidate, Junki Fukumoto, Aki Sato, Hideki Usuda, Komei Tanaka, Kazuyoshi Takahashi, Shirou Kuwabara

**Affiliations:** 1 Department of Digestive Surgery, Niigata City General Hospital, Shumoku, Chuo-ku, Niigata 463-7 Japan; 2 Department of Pathology, Niigata City General Hospital, Niigata, Japan; 3 Department of Neurology, Niigata City General Hospital, Niigata, Japan; 4 Department of Cardiology, Niigata City General Hospital, Niigata, Japan

**Keywords:** Gastric cancer, Nivolumab, Myocarditis, Myasthenia gravis, Programmed cell death-1 receptor

## Abstract

**Background:**

Although rare, several immune-related adverse effects can be life-threatening. Here, we describe a metastatic gastric cancer patient presenting with nivolumab-related myasthenia gravis and myocarditis, a previously unreported adverse effect of gastric cancer treatment.

**Case presentation:**

A 66-year-old man with metastatic gastric cancer visited the emergency department because of dizziness after the first dose of nivolumab. Diagnoses of nivolumab-related myasthenia gravis and myocarditis were established. Myocardial biopsy results and anti-acetylcholine receptor antibody positivity confirmed the diagnoses. Despite plasma exchange and intravenous methylprednisolone and immunoglobulin administration, the patient’s general condition gradually worsened, and he died.

**Conclusions:**

Strict monitoring for cardiac and neuromuscular symptoms after nivolumab administration is necessary to rapidly treat these adverse effects.

## Background

Immune checkpoint inhibitors (ICIs) such as nivolumab, known as anti-programmed cell death protein 1 (PD-1) inhibitors, are widely used to treat metastatic cancer patients. Anti-PD-1 antibody is used as a third-line treatment for unresectable, advanced, or recurrent gastric cancer. However, despite their beneficial effects in treating numerous tumors, ICIs can induce several immune-related adverse effects (irAEs) [1].

Adverse effects of nivolumab in metastatic gastric cancer patients were summarized in phase 3, ATTRACTION-2 clinical trial [2]. In the nivolumab group, all-grade treatment-related adverse events reported in 5% or more of patients were pruritus, diarrhea, rash, and fatigue. Moreover, this group also exhibited some serious treatment-related adverse events: interstitial lung disease (n = 3) and colitis, pyrexia, pneumonia, urinary tract infection, and diabetic ketoacidosis (n = 2 each). The incidence rates of myasthenia gravis (MG) and myocarditis as irAEs are less than 1%, with a mortality rate of 37.5% (3 out of 8) when they occur simultaneously (Table 1) [1, 3]. The co-occurrence of MG and myocarditis is a previously unreported adverse event after gastric cancer treatment. Here, we present a case of nivolumab-related MG and myocarditis occurring in a metastatic gastric cancer patient. We believe this is a crucial finding since gastric cancer is highly prevalent in Asia.

**Table 1:** Previously reported studies on nivolumab-induced myasthenia gravis and myocarditis

No	References	Malignancy	Year	Age	Sex	Regiment	After PD-1 cycle	CPK(IU/L)	Anti-striational antibodies	Steroid	IVIG	Plasma exchange	Intubation	Outcome
1	Kimura et al. [15]	Melanoma	2016	80	M	Nivolumab	1	9536	Not mentioned	+	+	+	−	Improve
2	Fukasawa et al. [16]	Lung cancer	2017	69	F	Nivolumab	3	1156	Not mentioned	+	−	−	NPPV	Improve
3	Fazel and Jedlowski et al. [17]	Melanoma	2019	78	F	Nivolumab + Ipilimumab	≧4	9198	+	+	+	+	−	Death
4	So et al. [18]	Melanoma	2019	55	F	Nivolumab	2	13,652	−	+	+	+	+	Improve
5	Rota et al. [19]	Renal cancer	2019	72	F	Nivolumab	1	Not mentioned	Not mentioned	+	+	−	−	Improve
6	Rota et al. [19]	Renal cancer	2019	71	M	Nivolumab	1	Not mentioned	Not mentioned	+	+	−	−	Death
7	Xing et al. [20]	Lung cancer	2020	66	M	Sintilimab	2	11,919	Not mentioned	+	+	+	+	Improve
8	Our case	Gastric cancer	2020	66	M	Nivolumab	1	8903	+	+	+	+	−	Death

*F* female; *M* male; *PD-1* programmed cell death 1; *CPK* creatine phosphokinase; *IVIG* intravenous immunoglobulins; *NPPV* noninvasive positive pressure ventilation; *yr* year

## Case presentation

**Fig. 1:**
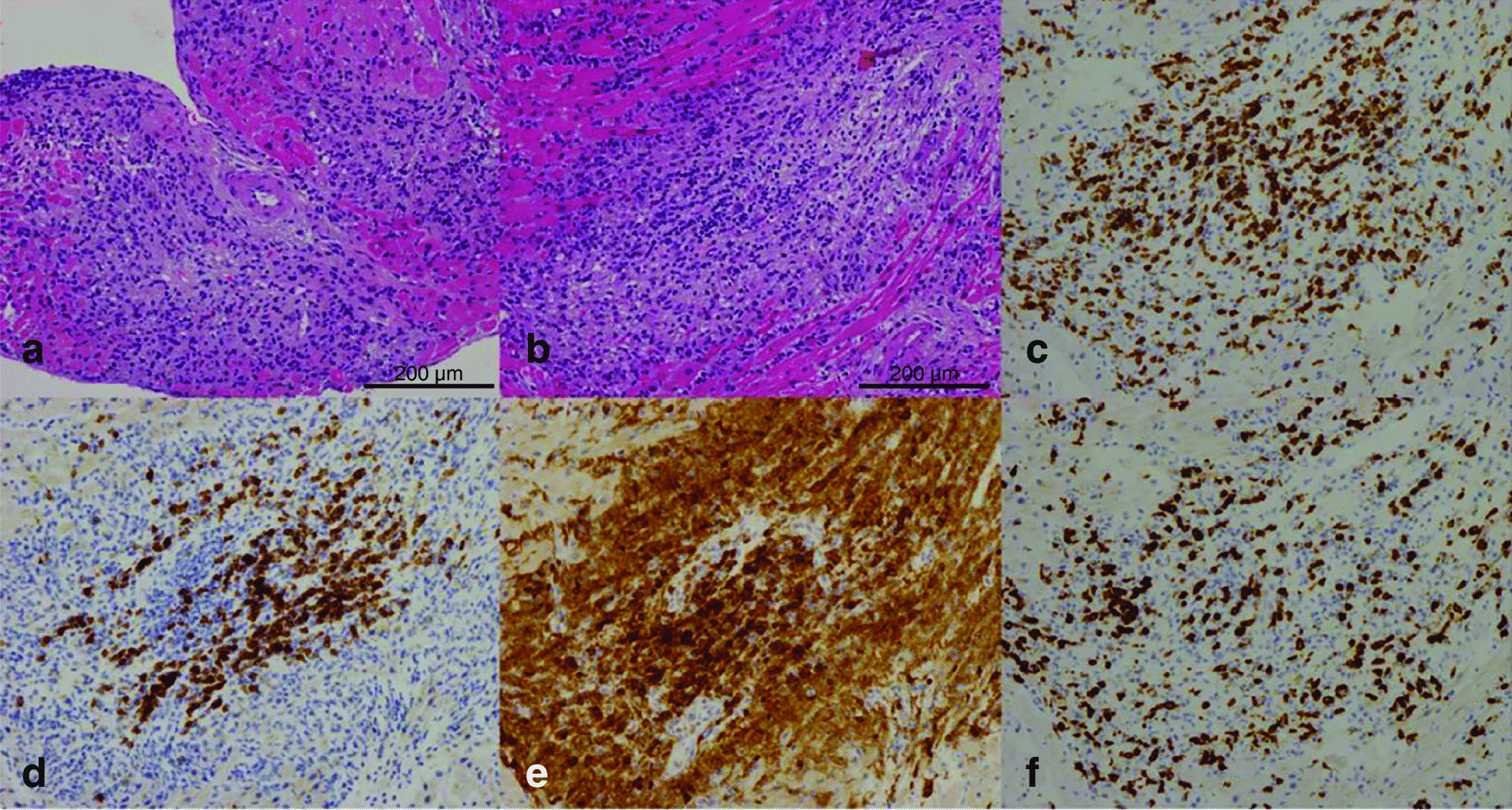
Pathological findings of myocardial biopsy; **a** Lymphocytic and macrophages infiltration. **b** Shedding of cardiomyocytes (30–40%) or severe degeneration is observed. **c**, **d** CD3-dominant T cells were observed than CD20. **e**, **f** CD8-dominant T cells were higher in number than CD4 cells. **a**, **b**: H & E, × 150; **c**: CD3, × 150; **d**: CD20, × 150; **e** CD4, × 150; **f** CD8, × 150

One month after the initial diagnosis of gastric cancer in a 66-year-old man, a laparoscopic examination revealed peritoneal dissemination, and a diagnosis of stage IV gastric cancer was made. His medical history did not include heart disease, neurological disease, or thymoma. Titanium silicate (TS-1) and cisplatin were selected as first-line treatments for advanced gastric cancer. Further, paclitaxel and ramucirumab were used as second-line treatments. Although the above treatment was performed, the patient appeared to have a progressive disease, and nivolumab alone was selected as the third-line chemotherapy. Twenty-four days after the first nivolumab infusion (240 mg/body) as third-line therapy, he experienced dizziness and difficulty breathing, which necessitated the visit to an emergency department. Laboratory evaluations demonstrated elevated levels of creatine phosphokinase (CPK) (8903 IU/L; normal range [NR]: 59–248 U/L), creatine kinase-MB (289 U/L; NR: 0–12 U/L), and troponin I (16,256 pg/mL; NR: 0–34.2 pg/mL). The alkaline phosphatase levels (171 U/L; NR: 106–322 U/L) and γ-glutamyl transpeptidase (14 U/L; NR: 13–64 U/L) were not elevated, and the patient had no symptoms of hepatotoxicity. Computed tomography showed no lesions in the brain. However, the patient presented with ventricular tachycardia even though there was no evidence of ischemia in coronary angiography, ruling out acute myocardial infarction. Myocardial biopsy demonstrated lymphocyte and macrophage infiltration, 30%–40% shedding of cardiomyocytes, and severe degeneration. Immunohistochemistry results demonstrated CD8 + T cells and macrophages within the myocardial tissue (Fig. 1). Thus, we diagnosed the patient with the irAE myocarditis.

The patient then developed progressive ophthalmoplegia, ptosis, dysphagia, dyspnea, and limb weakness. Repeated nerve stimulation revealed no waning, and anti-acetylcholine receptor (AchR) antibodies were detected in the serum. Thus, the diagnostic criteria of MG were met. We diagnosed the patient with MG, concomitantly with nivolumab-related myocarditis. The occurrence of concomitant myositis was not confirmed as muscle biopsy had not been performed. Blood test results for antibodies to muscle-specific kinase and low-density lipoprotein receptor-related protein 4 were negative. However, anti-striational antibodies, including antibodies against titin and muscular voltage-gated potassium channel 1.4, were positive. Pulse methylprednisolone (1.0 g/day) was initiated for 3 days after admission to treat nivolumab-related MG and myocarditis, followed by a dose of 1 mg/kg/day. On the seventh day after hospital admission, a Mobitz type II atrioventricular block was observed after electrocardiography, and a temporary cardiac pacemaker was implanted. The levels of CPK, CK-MB, troponin I, aspartate aminotransferase, and alanine aminotransferase gradually decreased.

**Fig. 2:**
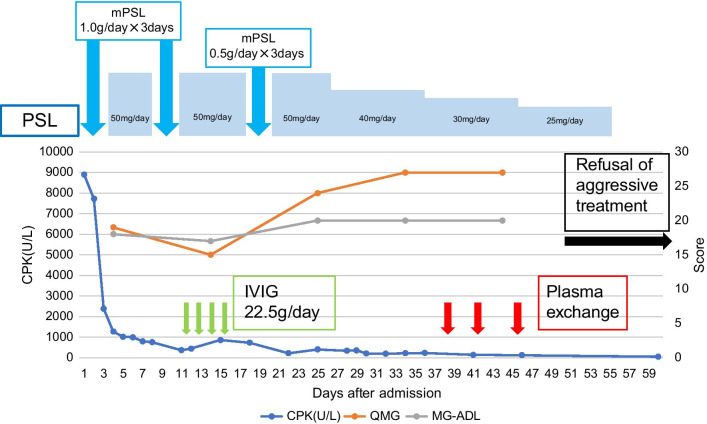
Clinical course. *PSL* prednisolone; *mPSL* methylprednisolone; *CPK* creatine phosphate kinase; *QMG* quantitative myasthenia gravis; *MG-ADL* myasthenia gravis activities of daily living, *IVIG* intravenous immunoglobulin

A high dose of intravenous methylprednisolone (1.0 g/day) was initiated; however, symptoms of MG worsened progressively. After 7 days of the initial infusion, an additional infusion of intravenous methylprednisolone (1.0 g/day) was administered. Intravenous immunoglobulins (IVIG) (22.5 g/day) were also administered. Subsequently, three plasma exchange cycles were completed. Despite plasma exchange and intravenous administration of methylprednisolone and immunoglobulins, the status of MG in the patient gradually worsened, and he died of type II respiratory failure due to progression of myasthenia gravis 103 days after admission. The clinical course is shown in Fig. 2.

## Discussion and conclusions

Gastric cancer accounts for 5.7% of all cancers globally. Although the incidence of gastric cancer is relatively low in Western countries, it is the highest in Eastern Asia, including Japan [4]. Gastric cancer is the third leading cause of death in Japan [5]. Nivolumab is recommended for stage IV gastric cancer patients as third-line therapy, based on the Japanese classification of gastric carcinoma [6].

Fulminant myocarditis related to ICIs has been reported; it occurs in < 1% of patients and has a 46% risk of death [1, 7]. ICI-related myocarditis often occurs after the first or second cycle of therapy [7]. Diagnosis of myocarditis is difficult because the sudden onset of heart failure or arrhythmias may reflect ischemic heart disease. Fundamentally, in ICI-related myocarditis, coronary angiography does not show abnormal findings. Ultimately, differential diagnosis of acute myocardial infarction is essential. Endo-myocardial biopsy is required for a definitive diagnosis, while histology will show mononuclear cell infiltration, rupture of cardiomyocytes, interstitial edema, and other similar findings.

In terms of irAEs, nivolumab-related MG and myocarditis have clinical characteristics different from those of idiopathic versions of the disease. In a study that included patients with melanoma and lung cancer, among other cancer types, Moslehi et al. [7] reported that concurrent irAEs, including myositis (25%) and MG (11%), occurred in 42 (42%) of 101 cases of ICI-related myocarditis. Typically, neurologic irAEs have been reported in < 1% of patients treated with ICIs [8]. Suzuki et al. [3] reported 12 MG cases (0.12%) among 9869 patients with cancer who were treated with nivolumab. Serum CPK concentrations are higher in nivolumab-related MG and concomitant myocarditis than in idiopathic MG [3]. Most reported cases of nivolumab-related MG rapidly deteriorated after 1 or 2 treatments with nivolumab [3]. Nivolumab-related MG tends to worsen respiratory status; thereby, requiring ventilatory support in 42% of cases. In contrast, it is required in 7% of idiopathic MG cases [3]. Patients with irAEs and preexisting immune diseases have higher frequencies of myasthenic crisis and myocarditis [7, 9, 10]. In a systematic review, 35% of MG patients associated with anti-PD-1 monoclonal antibodies had histories of preexisting MG [11]. Our patient had no history of immune disease. However, testing for anti-AchR antibodies may be useful in avoiding a fatal myasthenic crisis in patients with thymoma or immune disease. Additionally, myocarditis co-occurs more often with nivolumab-related MG rather than idiopathic MG. Myocarditis was found only in 0.3% of patients with idiopathic MG compared to 33% (4 of 12) of nivolumab-related MG patients [3]. Thus, care should be taken, especially early after nivolumab administration, as the co-occurrence of nivolumab-related MG and myocarditis rapidly worsens the patient’s condition and results in fatality.

Suzuki et al. [12] stated that anti-striational antibodies are useful clinical markers of developing myocarditis and that the heart can be an autoimmune target in some patients with MG. Moreover, severe outcomes such as death were more commonly observed in nivolumab-related MG with anti-striational antibodies than in seronegative cases [13]. Thus, anti-striational antibodies are possible biomarkers for irAEs because they are associated with myocarditis in patients with MG [3]. Skeletal muscles may also be autoimmune targets. Anti-striational antibodies were positive in our patient as well. However, it is unclear how much it affected our patient outcomes.

Most reports of ICI-related myocarditis have been in melanoma patients, followed by lung cancer and other types of cancer [7]. Likewise, patients presenting with nivolumab-related MG most commonly had lung cancer, melanoma, or other types of cancers [3], suggesting no difference in the incidence of ICI-related adverse events by cancer type [14]. However, an established theory on this relationship is still lacking.

This is the first report on the co-occurrence of nivolumab-related MG and myocarditis, especially in metastatic gastric cancer. The lack of such co-occurrences in gastric cancer may be because nivolumab is used less frequently in gastric cancer than in other cancers. In Japan, nivolumab was indicated for gastric cancer approximately 2 years later than malignant melanoma and lung cancer. The side effects similar to those in this case may increase with the increased use of nivolumab. Approximately half of the patients with ICI-related MG and myocarditis have died (Table 1). Thus, after administering nivolumab, clinicians should immediately consult a specialist if the patient has cardiac, muscular, or neurological symptoms. Clinicians should consider an early introduction of aggressive therapy, such as intravenous methylprednisolone, IVIG, plasma exchange, and respiratory support, in the treatment plan. Therefore, nivolumab should be used in general hospitals with specialty departments, such as cardiology and neurology; a delay in initial response may lead to poor outcomes.

We reported a rare case of nivolumab-related MG and myocarditis in a metastatic gastric cancer patient. This report demonstrates the importance of improving the understanding of nivolumab-related MG with concomitant myocarditis after nivolumab administration to improve clinical outcomes.

## Data Availability

The datasets used and/or analyzed during the current study are available from the corresponding author on reasonable request.

## Abbreviations

AchR: Acetylcholine receptor
CK: Creatine kinase
CPK: Creatine phosphokinase
ICI: Immune checkpoint inhibitor
irAEs: Immune-related adverse effects
IVIG: Intravenous immunoglobulin
MG: Myasthenia gravis
NR: Normal range
PD-1: Programmed cell death protein 1
